# RETRACTED: Xin et al. Dexras1 Induces Dysdifferentiation of Oligodendrocytes and Myelin Injury by Inhibiting the cAMP-CREB Pathway After Subarachnoid Hemorrhage. *Cells* 2022, *11*, 2976

**DOI:** 10.3390/cells15212021

**Published:** 2026-11-06

**Authors:** Yuanjun Xin, Jie Chen, Hongxia Zhang, Robert P. Ostrowski, Yidan Liang, Jun Zhao, Xiang Xiang, Fuming Liang, Wenqiao Fu, Hao Huang, Xintong Wu, Jun Su, Jiewen Deng, Zhaohui He

**Affiliations:** 1 Department of Neurosurgery, The First Affiliated Hospital of Chongqing Medical University, 1 Friendship Road, Chongqing 400016, China; 2 Laboratory of Skeletal Development and Regeneration, Institute of Life Sciences, Chongqing Medical University, Chongqing 400016, China; 3 Department of Experimental and Clinical Neuropathology, Mossakowski Medical Research Institute Polish Academy of Sciences, 02-106 Warsaw, Poland

The journal retracts the article titled “Dexras1 Induces Dysdifferentiation of Oligodendrocytes and Myelin Injury by Inhibiting the cAMP-CREB Pathway after Subarachnoid Hemorrhage” [1], cited above.

Following the publication of a correction [2], additional concerns were brought to the attention of the Editorial Office regarding the integrity of several figures presented in the publication.

In accordance with the journal’s standard procedures, the Editorial Office and the Editorial Board conducted an investigation that identified indications of duplication between Figures 6E and S1B, Figures 2B and 3A, and within Figure 3 (3D Dexras1/Iba-1/DAPI and 3F SAH Iba-1/DAPI). Although the authors cooperated with the investigation, the explanations and supporting materials provided were not deemed sufficient by the Editorial Board to resolve the identified concerns. Consequently, the Editorial Board has lost confidence in the reliability of the reported findings and has decided to retract this publication in accordance with MDPI’s retraction policy.

This retraction was approved by the Editor-in-Chief of *Cells*.

The authors have been informed of this retraction and have indicated their agreement with this decision. The authors state that the issues arose during figure preparation.
